# A dynamic model of gene activation in response to hypoxia accounting for both HIF-1 and HIF-2

**DOI:** 10.1093/bfgp/elaf021

**Published:** 2025-12-12

**Authors:** Aleksandra Cabaj, Agata Charzyńska, Adrianna Moszyńska, Maciej Jaśkiewicz, Rafał Bartoszewski, Michał Dąbrowski

**Affiliations:** Laboratory of Sequencing, Nencki Institute of Experimental Biology, Pasteura 3 Street, Warsaw 02-093, Poland; Laboratory of Language Neurobiology, Nencki Institute of Experimental Biology, Pasteura 3 Street, Warsaw 02-093, Poland; Department of Biophysics, Faculty of Biotechnology, University of Wroclaw, F. Joliot-Curie 14a Street, Wroclaw 50-383, Poland; Laboratory of Photobiology and Molecular Diagnostics, Intercollegiate Faculty of Biotechnology, University of Gdansk and Medical University of Gdansk, Abrahama 58 Street, 80-307 Gdansk, Poland; Department of Biophysics, Faculty of Biotechnology, University of Wroclaw, F. Joliot-Curie 14a Street, Wroclaw 50-383, Poland; Laboratory of Molecular Neurobiology, Nencki Institute of Experimental Biology, Pasteura 3 Street, Warsaw 02-093, Poland

**Keywords:** hypoxia, HIF-2, dynamic ODE model, hypoxia response element, promoter, HIF-1β alias ARNT

## Abstract

We developed an ordinary differential equations (ODEs) model of hypoxia signaling that, in addition to HIF-1α, takes into account also HIF-2α. Our model can be separated into two parts, the first, describing the production and degradation of the α subunits of HIF-1 and HIF-2, and their accumulation in response to hypoxia; and the second, describing how the α subunits cooperate with the β subunit in binding to cis-regulatory regions and activation of HIF-target genes in response to hypoxia. In our previous work [1], using the first part of our model trained on time-series data from 0.9 % hypoxia, we successfully predicted the response of the system to a further drop of the oxygen to 0.3 % hypoxia. This modeling result contributed to explaining the mechanism of the switch of the control from HIF-1 to HIF-2 during the response of human primary endothelial cells to hypoxia. In another work [2], we experimentally demonstrated a linear proportionality between the counts of motifs assigned to HIF-1 in promoter open chromatin regions of genes and the effects of HIF-1 on the induction of these genes under hypoxia. We furthermore showed that such a proportionality is predicted by the subset of the ODE model of Nguyen et al. (2013) [3] common with the second part of our ODE model. In the current work, we provide the details of our full ODE model and show that it leads to a prediction that HIF-1β can be a limiting factor of the response to hypoxia.

## Introduction

The response to hypoxia is among the best experimentally studied transcriptional responses to a stimulus acting on the cell, and therefore it is well suited for mathematical modeling. Cellular response to hypoxia is regulated transcriptionally by activation of hypoxia inducible factors (HIFs) 1 and 2 [4, 5]. HIF-1 and HIF-2 are heterodimers, composed of an inducible oxygen-sensitive α subunit (HIF-1α, encoded by *HIF1A;* or HIF-2α, encoded by *EPAS1,* alias *HIF2A;* respectively), and a common, constitutively expressed β subunit (HIF-1β), encoded by *ARNT*, alias *HIF1B* [6, 7]. For clarity of the ODE model equations and diagrams, and easy cross-reference between the text and the model, in the remaining text we refer to the proteins by the names of their encoding genes typed in plain font, e.g.: HIF1A denotes the HIF-1α protein, HIF2A denotes HIF-2α, etc., whereas the names of the corresponding mRNAs/genes are in italics: *HIF1A*, *HIF2A*, etc. Inside the model, where the distinction between italic and plain fonts is not used, we use suffixes (_protein, _mrna), to distinguish proteins from mRNAs. Additionally, inside the model HIF-1 and HIF-2 heterodimers are denoted HIF1AB and HIF2AB, respectively. HIF-1 induces the expression of glycolytic genes [8], pro-angiogenic genes, and genes involved in pH regulation [9], and promotes cellular metabolic adaption to the decreased oxygen levels [10]. HIF2A is expressed in specific cell types, including endothelial cells, cardiomyocytes, and hepatocytes [11]. HIF-2 facilitates expression of matrix metalloproteinases and erythropoietin and is required for the proper maturation of the vascular network [5, 12]. Under normal oxygen tension, HIF α subunits are rapidly post-translationally hydroxylated by specific HIF prolyl-hydroxylases (PHDs) [13, 14]. The prolyl-hydroxylation of HIF α subunits leads to their recognition by von Hippel–Lindau tumor suppressor protein, a component of an E3 ubiquitin ligase complex, and results in HIF α polyubiquitination and rapid degradation [15, 16]. During hypoxia, the HIF α subunits are stabilized and accumulate at the protein level. Subsequently, they are translocated to the nucleus, where they form heterodimers with the constitutive β subunit HIF1B [6]. In the nucleus, the HIF heterodimers bind to hypoxia response elements (HREs) in cis-regulatory regions (promoters and enhancers) of genes, leading to induction of hypoxia-responsive genes [17]. HIF-1 and HIF-2 bind to HRE sites containing the same core consensus sequence 5’-RCGTG-3′ [18, 19]. HIF2A has an additional N-TAD domain, not present in HIF1A, which contributes to differences in their DNA binding preferences [20]. In vivo, in addition to HRE sites bound by both HIFs, there are numerous HRE sites bound selectively by either HIF-1 or HIF-2. Separate analysis of these HRE sites led to identification of distinct but highly similar HRE motifs for HIF-1 and for HIF-2 [17, 21]. The results of the count analysis of these two motifs for genes induced with different kinetics under hypoxia suggest that there is a preference of HIF-1 and HIF-2 to their respective annotated motifs [22]. This is more important than one would suspect, because a careful comparison of the results of two previous studies [17, 21] reveals that both HIFs can bind to both motifs. In both cancer cells and in endothelial cells, HIF1A accumulates earlier during hypoxia and its levels decrease more rapidly than HIF2A during prolonged hypoxia [5, 22–24]. This results in a transition from HIF-1 to HIF-2 specific effects, which is called the HIF switch [25]. Although numerous factors have been proposed to contribute to the HIF switch [23, 26], the mechanism underlying the HIF1A elimination during prolonged hypoxia remains poorly understood.

The cellular response to hypoxia by the accumulation of HIF1A protein has been extensively modeled before with ordinary differential equations (ODEs), and several refined ODE models describing the cascade of events leading to the transcriptional activation of hypoxia inducible genes have been published [3, 27, 28]—reviewed in [29, 30]. A common feature of the above models is that they include only one isoform of the α subunit of the HIF transcription factor, namely the HIF1A protein, encoded by the *HIF1A* gene. Analysis of the HIF switch is not possible in the published ODE models describing the entire cascade of response to hypoxia, because they take into account only one isoform of the HIF α subunit, namely HIF1A. More recently, two ODE models have been published, which take into account both HIF1A and HIF2A [31, 32]. However, these models analyze some specialized processes and interactions, in which these two HIF α proteins are involved. Zhang et al. (2018) [31] analyzed interactions between either HIF1A or HIF2A and MYC, and their roles in hypoxia-mediated cell cycle commitment. Ferrante et al. (2023) [32] analyzed the interactions among HIF-1, HIF-2, PHD2, PHD3, and NFKB and their role in hypoxia-related inflammation. Neither of the above two models can be applied to modeling the HIF switch. Therefore, in order to model this phenomenon we developed and implemented our own ODE model of activation of HIF-1 and HIF-2 transcription factors during hypoxia.

Our full ODE model of hypoxia response can be divided into two parts. The first part describes the processes from the transcription of genes encoding HIF1A, HIF2A, and PHD, to accumulation of the two HIF α subunits under hypoxia. The second part describes the processes from the accumulation of HIF α subunits, through their dimerization with HIF1B, to the transcriptional activation of target genes. We refer to these parts as “cytoplasmic” and “nuclear”, respectively. To avoid confusion, we stress that these are names of the model parts, and not of separate compartments, so our model, similarly to that of Nguyen et al. (2013) [3], is a single compartment model. In our work published in CMBL [1], we used the cytoplasmic part of our ODE model to simulate the HIF switch. The results of the simulation were consistent with the results of the experiment. This supported the conclusion of that work, about the key role of the residual PHD2 activity under hypoxia and of the differences in the stability of mRNAs encoding HIF1A and HIF2A, for the dynamics of the HIF switch. In our work published in Cellular Signalling [2], by studying a carefully pre-selected set of genes, and by integrating genetic (sequence) and epigenetic (open chromatin) data, we experimentally demonstrated a linear proportionality between the count of HRE1 motifs, assigned to HIF1A, in the cis-regulatory region of a given gene and the effect of HIF1A on the induction of this gene under hypoxia. In the Supplementary Note to that publication, we showed that such a proportionality follows from a subset of the model of Nguyen et al. (2013) [3], which is also a subset of our full ODE model with HIF-2-related terms dropped. Below, we present in detail the structure of our full ODE model and its fitting to the data. We then present the results of simulations performed in this model, indicating that there is the need for a large excess of HIF1B, relative to the concentrations of the two HIF α subunits, for the model to reproduce the observed dynamics of the transcriptional activation of *MIR210HG*, whereas lower initial concentrations of HIF1B are required to mimic in the model the observed induction of two additional genes regulated by both HIF-1 and HIF-2. Our modeling results support the hypothesis, originally put forward by Mandl et al. (2013) [33], that HIF1B can be a limiting factor of the response to hypoxia, and permit a more specific prediction that this mechanism will affect primarily the genes utilizing many HRE sites strongly induced by hypoxia, such as *MIR210HG*.

## Materials and methods

### Sources of data used for model fitting

*Western blot and RT-qPCR data.* The data used as an input to fit our model to comprises of relative HIF1A, HIF2A, and PHD2 protein concentrations and *HIF1A* and *EPAS1* (*HIF2A*) mRNA relative expression. For each of these molecular species we had quantitative time-series data on their relative abundance at 12 time-points over 48 hours from Human Umbilical Endothelial Cells (HUVECs) under 0.9 % hypoxia [22, 24]. The changes in protein levels were evaluated by western blot densitometry and normalized to β-actin and total protein levels and expressed as a relative fold change over normoxic condition (0 h). mRNA levels were quantified by qRT-PCR in three replicates, normalized to *RPLP0* mRNA and 18S rRNA levels, and expressed as a fold change over normoxic samples (0 h). The above data we used before for fitting the cytoplasmic part of our ODE model, described in our CMBL work [1], and are available as “Supplementary File 1a” to that publication at https://doi.org/10.1186/s11658-022-00408-7.

*HRE motifs counts in promoter open chromatin regions.* We focused on the promoter open chromatin regions, defined as the DNase-hypersensitive regions of the HUVECs established by the ENCODE consortium [34] within ±10 kb flank of the gene start. The two ENCODE DNase I-seq datasets from HUVECs under normoxia found in Ensembl [35] were merged. We used HRE motifs: M00139 annotated to *HIF1A*, and M00074 annotated to *EPAS1*; from HOCOMOCO v. 9 [36, 37]. We used the Nencki Genomics Database [38] to obtain the genomic coordinates (hg38) of the instances of the two HRE motifs. Separately for either motif, we calculated the motif count per gene, as its cumulative count in all DHS regions within the flank. If a DHS region spanned a flank boundary, only the motifs within the flank were counted. These count data were used before in our Cellular Signalling work [2], and are available in “Appendix A. Supplementary data” to that publication at https://doi.org/10.1016/j.cellsig.2021.110209.

*HIF-target genes relative expression during hypoxia.* We furthermore used the relative mRNA expression values from the microarray experiment, at 2 h, 8 h, and 16 h of 0.9 % O_2_, also performed in HUVECs, normalized to normoxic levels (0 h) [22] for the 13 hypoxia-responsive genes we studied before in our Cellular Signalling work [2].

### Measuring and scaling of the concentrations of HIF α subunits

Measurements of the absolute concentrations of HIF1A and HIF2A were performed in duplicate, under normoxia, and at 2 h and 8 h of 0.9 % O_2_ hypoxia for HIF1A, and at 8 h and 24 h of 0.9 % O_2_ hypoxia for HIF2A. Cell lysis and further ELISA was performed using Human HIF-1-alpha ELISA Kit (Abcam, ab171577), and Human HIF-2-alpha ELISA Kit (Abcam, ab227898) according to the manufacturer’s protocols. Total protein for the assay was 25 μg / 50 μl sample for HIF1A and 12.5 μg / 50 μl sample for HIF2A. The absorbance was measured at 450 nm. We used the absolute concentration of HIF1A at 2 h, and the absolute concentration of HIF2A at 8 h, expressed as ng/mg of total protein, together with their relative fold changes over the normoxic condition (0 h), as measured by the western blots, to calculate the absolute concentrations of the two proteins at every time-point. These calculations were performed as follows:

HIF1A _absolute_ (2 h) / HIF1A _relative_ (2 h) = k_1_

HIF2A _absolute_ (8 h) / HIF2A _relative_ (8 h) = k_2_

HIF1A _relative_ (t) · k_1_ = HIF1A _absolute_ (t)

HIF2A _relative_ (t) · k_2_ = HIF2A _absolute_ (t)

Then, we divided the absolute concentration of HIF2A at 0 h (normoxia) by the absolute concentration of HIF1A at 0 h, to obtain the scaling constant m, which was then used to compute, for every time-point, the scaled relative concentration of HIF2A:

HIF2A _absolute_ (0 h) / HIF1A _absolute_ (0 h) = m

HIF2A _relative_ (t) · m = HIF2A _scaled_ (t)

### The ODE modeling environment

Our model was implemented in Matlab (v. R2017b) using the Simbiology package (v. 5.7) [39] along with the fitting of the model parameters to the experimental data and simulations. All Simbiology’s settings were set to default, unless stated otherwise. For every data fitting, it was assumed that normoxia is the initial condition. All fittings were performed with the same initial values of parameters, where we set all the initial reaction constants to 1, and all the initial dissociation constants (*.kd) to 10^-4^.

## Results

### Scaling the relative concentrations of HIF α subunits

In our previous studies we examined both biochemical responses and HIF-1- and HIF-2-related transcriptional activity during a hypoxia time course, particularly at 2 h, 8 h, and 16 h [1, 22]. As shown in Bartoszewski et al. (2019) [22], HIF1A begins to accumulate early during hypoxia (starting at 2 h) but becomes undetectable after 12 h. In contrast, HIF2A accumulation starts later (around 4 h) and persists for over 24 h.

**Figure 1:**
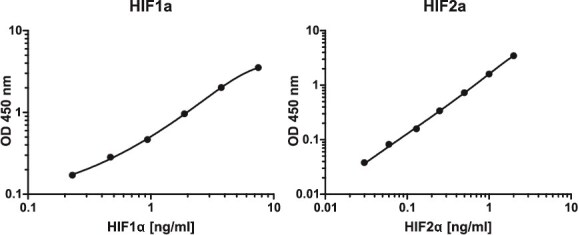
Standard curves of HIF1A and HIF2A in the ELISA assay—dependence between the optical density (OD) at 450 nm and the concentration of a given HIF α.

In the current work, using the standard curve approach (Fig. 1), we performed ELISA measurements of absolute concentrations of HIF1A at 0 h, 2 h, and 8 h under hypoxia; and of HIF2A at 0 h, 8 h and 24 h under hypoxia. The results of these measurements, expressed as ng/mg of total protein, are shown in Table 1. We chose the 2 h time-point, specific for the induction of HIF1A, to calculate the ratio between the absolute and the relative abundance of HIF1A; and the 8 h time-point, close to the time of the maximal induction of HIF2A, to calculate the ratio between the absolute and the relative abundance of HIF2A. These two ratios were used, as described in Materials and methods, to compute the ratio of the absolute concentration of HIF2A to the absolute concentration of HIF1A, both at normoxia (0 h). This ratio, the calculated value of which was 5.26, was then used as the scaling constant to compute the scaled relative concentration of HIF2A.

**Table 1:** Absolute concentrations of HIF1A and HIF2A measured with ELISA and calculated based on the standard curves shown in Fig. 1. The values used to compute the HIF2A protein scaled are shown in bold.

**HIF1A**						
	OD 450 nm abs1	OD 450 nm abs2	OD 450 nm average	OD 450 nm average − blank	HIF1A [ng/ml]	**HIF1A** **[ng/mg of total protein]**
normoxia	0.17	0.172	0.171	0.123	0.098	0.196
**hypoxia 2 h**	0.24	0.237	0.239	0.191	0.273	**0.546**
hypoxia 8 h	0.258	0.266	0.262	0.214	0.330	0.660
**HIF2A**						
	OD 450 nm abs1	OD 450 nm abs2	OD 450 nm average	OD 450 nm average − blank	HIF2A [ng/ml]	**HIF2A** **[ng/mg] of total protein**
normoxia^#^	0.068	0.068	0.068	0.020	0.017^#^	0.068^#^
**hypoxia 8 h**	1.418	1.388	1.403	1.355	0.867	**3.468**
hypoxia 24 h	1.14	1.135	1.138	1.090	0.717	2.868

^#^The very low measured value of the absolute concentration of HIF2A at normoxia is not credible, given the absolute measurements at the two hypoxic time-points and the relative abundance data (Table 2). Using these data, indicating 5.56-fold induction of HIF2A between 0 h and 8 h, and its absolute concentration of 3.47 ng/mg at 8 h, we calculate the absolute normoxic concentration of HIF2A as 0.62 ng/mg.

### Complete data used for ODE model fitting

The complete data used for ODE model fitting, originating from our publications [1, 2, 22] are given in Table 2 (time-series data). For fitting our full ODE model we used the scaled relative concentration of HIF2A, to better account for competition between the two HIF α subunits for the β subunit, and between HIF-1 and HIF-2 for the HREs.

**Table 2:** Time-series input data used for the model fitting. All of the values are non-logarithmic and represent relative concentrations of those molecules in relation to normoxic levels (0 h), apart from “HIF2A protein scaled”, which is additionally scaled by the ratio of the absolute concentration of HIF2A to the absolute concentration of HIF1A, both at 0 h.

time (hours)	HIF1A protein	HIF2A protein	HIF2A protein scaled	PHD protein	*HIF1A* mRNA	*HIF2A* mRNA	*MIR210HG* mRNA
0	1.0000	1.0000	5.2606	1.0000	1.0000	1.0000	1.0000
2	4.6080	1.9469	10.2416	1.2631	0.5800	0.6475	6.8630
4	6.2155	2.3412	12.3161	1.3703	0.6100	0.7841	
6	8.4539	5.1084	26.8728	1.8831	0.4000	0.7462	
8	7.6271	5.5638	29.2685	2.1122	0.2600	0.5248	40.6453
10	5.8307	6.7598	35.5602	2.6777	0.2500	0.8267	
12	4.7802	6.5332	34.3684	2.5870	0.2500	0.5701	
16	3.0066	6.5175	34.2855	3.6042	0.1300	0.5221	46.2323
20	3.0746	5.5817	29.3627	3.5460	0.2600	0.7198	
24	2.1006	4.3697	22.9869	4.3130	0.3600	0.8510	
36	2.4858	4.7546	25.0117	5.7481	0.4100	0.8653	
48	1.3927	3.5150	18.4910	6.2099	1.0500	1.8974	

### Model equations and design

**Figure 2:**
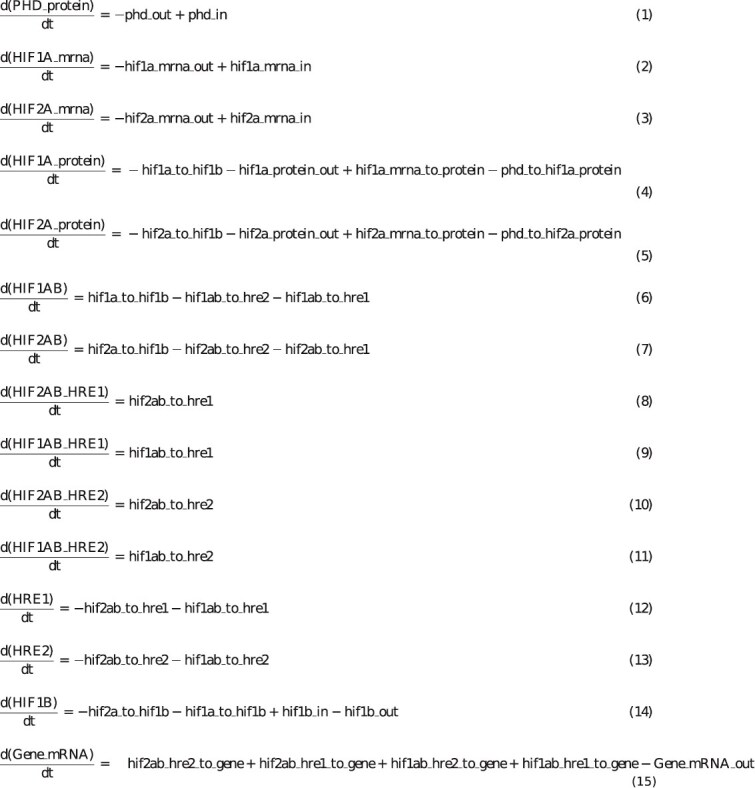
Ordinary differential equations of the model.

Our model consists of 15 ODEs—one for each of the key molecular species or complexes participating in the response to hypoxia in HUVECs. These equations are shown in Fig. 2, with the corresponding reactions and reaction rates (fluxes) shown in Suppl. Table S1. In our model, the key reaction rate of HIF1A degradation due to its hydroxylation by PHD (Suppl. Table S1, reaction 1) follows the established model of HIF1A protein signaling of Nguyen et al. (2013) [3]. In that model, the reaction of HIF1A hydroxylation by PHD is described by the Michaelis-Menten kinetics, with oxygen and HIF1A as the two substrates. We designed our model to include also HIF2A, by adding an analogous reaction of HIF2A degradation due to hydroxylation by PHD (Suppl. Table S1, reaction 2). The remaining reactions were modeled as first-order reactions using the Mass-Action Law [40, 41]. We assumed that the system contains a single dominant PHD isoform, which can have potentially different activities towards HIF1A and HIF2A, in agreement with the available experimental evidence coming partially from endothelial cells [42, 43]. Both HIF1A and HIF2A can bind to HIF1B, resulting in formation of HIF1AB and, respectively, HIF2AB heterodimers (Suppl. Table S1, reactions 13–14).

In addition to modeling the HIF switch, our goal when developing the ODE model of cellular response to hypoxia was to include in this model gene regulation not only by HIF-1, but also by HIF-2, or both HIFs. We did it by including into our model a generic HIF-target gene, or a target gene for short, which is regulated via HRE motifs assigned to HIF-1, named HRE1, and via HRE motifs assigned to HIF-2, named HRE2. Either HRE type can bind both HIF-1 and HIF-2, with separate affinities, and we explicitly allow such cross-binding in the model (Suppl. Table S1, reactions 15-18). We furthermore assume that the transcriptional rate of the target gene is proportional to the amounts of HIF-1 and HIF-2 bound to HRE motifs (Suppl. Table S1, reactions 19-22). We treat the counts of HRE motifs in promoter open chromatin regions as their initial concentrations, which is justified by the proportionality we demonstrated in [2].

### Model overview

**Figure 3:**
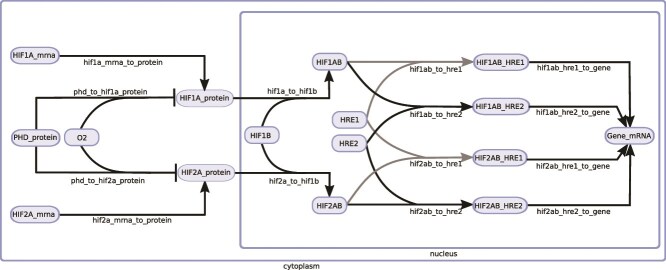
A simplified diagram of our model of hypoxia signaling taking into account both HIF-1 and HIF-2.

A simplified diagram of our model of the response to hypoxia providing an overview of its key features is shown in Fig. 3. Blue ovals represent the model species (e.g. HIF1A_mrna) and arrows represent reactions between the species (e.g. hif1a_mrna_to_protein). The left part of the diagram illustrates the cytoplasmic part of the model, describing mRNAs species being translated to HIF proteins (hif1a_mrna_to_protein and hif2a_mrna_to_protein) and the simplified process of HIF degradation due to oxygen-dependent PHD-mediated hydroxylation of those proteins (phd_to_hif1a_protein and phd_to_hif2a_protein). The central part of the diagram illustrates that in our model HIF1A and HIF2A can reversibly dimerize with HIF1B to form two types of transcriptionally active HIF heterodimers, namely HIF1AB and HIF2AB, respectively. We assume that a target gene can contain certain numbers of two types of HRE motifs, designated HRE1 and HRE2. We further assume that each type of HRE motif can reversibly bind both types of HIF heterodimers (with possibly different affinities), resulting in four possible HIF-HRE complexes (e.g. HIF1AB_HRE2). Finally, we assume that each type of HIF-HRE complex can activate (possibly with different effectiveness) the transcription of the target gene. For a complete diagram of the model (Supplementary Figure S1) and its description see Supplementary File 1.

**Figure 4:**
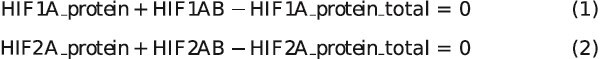
Algebraic rules describing the relationship between the HIF α subunits, the HIF heterodimers and the total relative amount of HIF proteins in the system.

In western blots we cannot distinguish the free HIF-1α and HIF-2α subunits from these subunits contained in HIF-1 and HIF-2 heterodimers. In our model (Fig. 3, Suppl. Fig. S1), HIF1A_protein and HIF2A_protein represent the free HIF-1α and HIF-2α subunits, whereas HIF1A_protein_total and HIF2A_protein_total (Suppl. Fig. S1) represent the sum of the respective free HIF α subunit and of this HIF α subunit bound to HIF-1β. The relationship between these three types of species is described with two simple algebraic rules shown in Fig. 4.

### Fitting of the full ODE model

The fitting of our full ODE model was performed in two steps. In the first step, performed as described in our publication [1], the cytoplasmic part of the model was fitted to the data in Table 2, using the relative concentrations of the molecular species represented in the cytoplasmic part of the model. In the second fitting step, the full model was fitted to the data in Table 2, using the scaled relative concentration of HIF2A, and the relative concentrations of the remaining molecular species, for the initial conditions given in Table 3. During this step, only the parameters of the reactions in nuclear part, and of the reactions in the cytoplasmic part involving HIF2A (hif2a_mrna_to_protein, hif2a_protein_out, phd_to_hif2a_protein), were allowed to change. The remaining parameters were fixed at their values fitted during the first step.

**Table 3:** Initial conditions of the final model.

Species	Initial conditions
HIF1AB	0
HIF2AB_HRE1	0
HIF1AB_HRE1	0
Gene_mRNA	1
HIF2AB_HRE2	0
HIF1AB_HRE2	0
HIF2AB	0
HIF1B	60
PHD_protein	1
HIF1A_mrna	1
HIF2A_mrna	1
HIF1A_protein	1
HIF2A_protein	5.26
HIF2A_protein_total	1
HIF1A_protein_total	1
O_2_	1
*MIR210HG* HREs:	HRE1: 12
	HRE2: 18

### Finding the optimal HIF1B initial concentration

**Figure 5:**
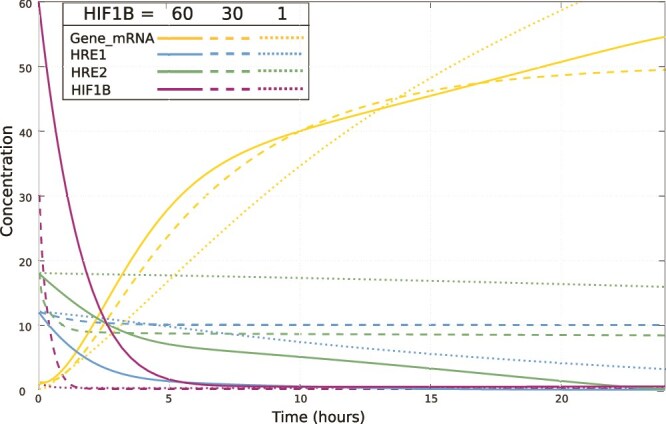
Simulations of the target gene expression (*MIR210HG*) and of the concentrations of the free (unbound) HIF1B, HRE1 and HRE2; in three separate model fits, for three different relative concentrations of HIF1B set as the initial condition.

**Figure 6:**
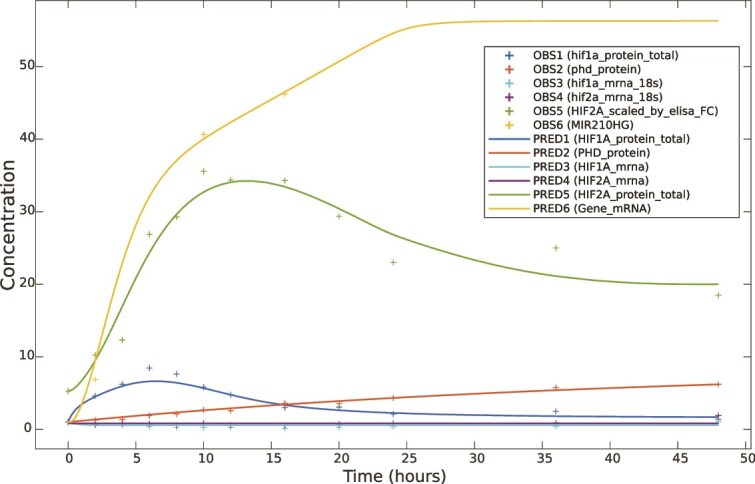
The result of fitting the final model to experimental data and *MIR210HG* target gene. The predicted values are shown as continuous lines and the observed values are represented as crosses. The X axis represents the time of the simulation in hours, and the Y axis shows the relative concentration of the species included in the model.

Because of a possible competition between α subunits for the β subunit and because the absolute concentration measurement of HIF1B was not available to us, it was important to choose an appropriate HIF1B initial concentration. We did it by fitting the full model for several initial concentrations of HIF1B (Fig. 5), and the remaining initial conditions as in Table 3. In the absence of experimental evidence on HIF1B concentration, we allowed its inflow and outflow to be fitted to the data. We started by setting the initial concentration of HIF1B to 1 (Fig. 5, dotted lines), the same as the initial concentration of HIF1A. In the model fitted for the initial concentration of HIF1B equal to 1, the induction of the target gene mRNA (Fig. 5, yellow dotted line) was more delayed than the induction of *MIR210HG* observed experimentally (Table 2 and Fig. 6). In the model fitted for the initial concentration of HIF1B set to 30, the response of the *MIR210HG* target gene was close to that observed experimentally (Fig. 5, yellow dashed line). However, at the initial concentration of 30 HIF1B was still a limiting factor, with the concentrations of free HRE1 and HRE2 reaching stationary levels, indicating utilization of two out of 12 HRE1 sites and half of 18 HRE2 sites of *MIR210HG* (Fig. 5, dashed lines)*.* These stationary levels of occupation of HRE1 and HRE2 sites were reached at 6 h under hypoxia, at which time the combined concentration of HIF1A and HIF2A (scaled) reached approx. 35 (Table 2), well before the maximum of their combined concentration (approx. 41) reached at 10 h under hypoxia. Only in the model fitted for the initial concentration of HIF1B set to 60 all the HRE sites became occupied (Fig. 5, continuous lines). For the above reasons, we chose the model fit for the initial concentration of HIF1B set to 60 as our final model for the *MIR210HG* target gene. The results of this fit, for the initial conditions in Table 3, are shown in Fig. 6. For all the measured species the obtained fit is remarkably good, considering the size and the complexity of the model.

### Testing the model for other target genes and concentrations of HIF1B

**Figure 7:**
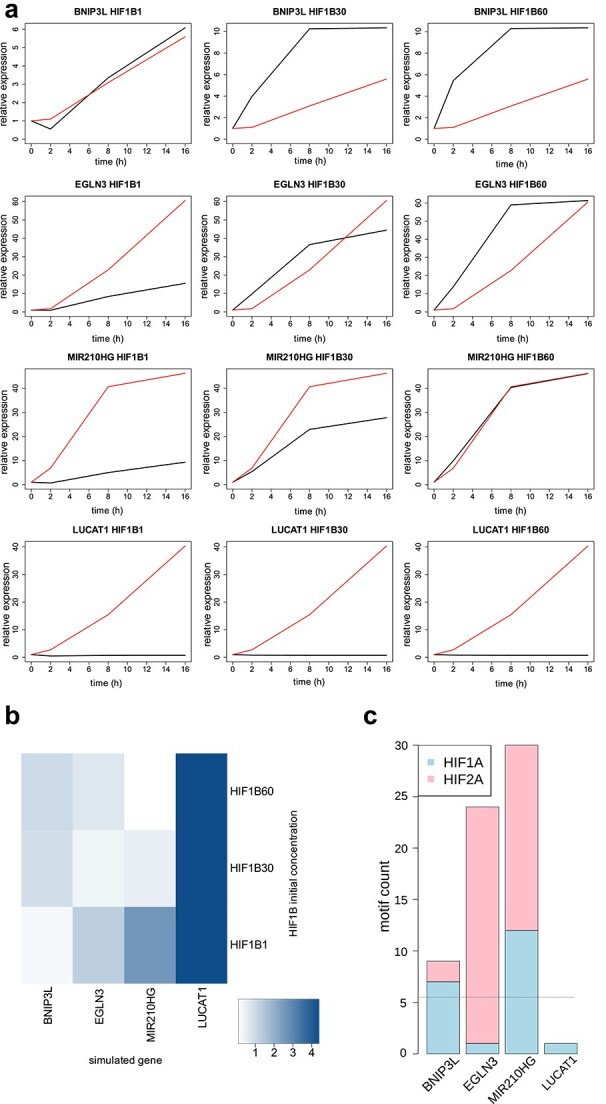
Predictions from the simulations in the final model compared to the observed expression, for the indicated genes: *BNIP3L*, *EGLN3*, *MIR210HG,* and *LUCAT1,* and the initial HIF1B concentration in the simulation set to: 1 (HIF1B1), 30 (HIF1B30), or 60 (HIF1B60). (a) The observed expression (red), and the expression predicted from the simulation (black), at 4 time-points during hypoxia: 0 h (normoxia), 2 h, 8 h, 16 h. (b) Goodness of fit, represented as a heatmap of the absolute(log2(average over the 4 time-points(ratio between the observed and the predicted expression))). (c) The counts of HRE1, HRE2, and the total HRE count, in promoter open chromatin regions.

We were interested if our final model, fitted for *MIR210HG* and the initial concentration of HIF1B set to 60, would be able to predict induction under hypoxia of additional genes among 13 hypoxia-responsive genes characterized in our Cellular Signalling work [2]. In that work, by using specific siRNAs*,* we identified four genes, including *MIR210HG* and three additional genes: *BNIP3L*, *EGLN3*, and *LUCAT1,* as regulated in HUVECs by both HIF1A and HIF2A, whereas the remaining nine genes were regulated either by HIF1A only or by HIF2A only, despite containing both HRE1 and HRE2 in their promoter open chromatin regions. We thus conclude that these nine genes were regulated by mechanisms governing their specificity for a particular HIF α, which are not reflected in our ODE model. We therefore decided to utilize only the three additional genes we characterized as regulated by both HIFs for testing the ability of our final model to predict induction of other genes under hypoxia. Additionally, for these genes and also *MIR210HG*, we analyzed the effects of different initial concentrations of HIF1B (1, 30, 60) on the simulations in the final model. The observed expression of these genes at 0 h, 2 h, 8 h, and 16 h of 0.9% hypoxia, and their predicted expression at these time-points obtained from the simulations, for the three different initial concentrations of HIF1B, are shown in Fig. 7 A. The goodness of fit between the observed and predicted expression, for every target gene and initial HIF1B concentration, are shown as a heatmap in Fig. 7 B, and the HRE counts are given in Fig. 7 C. As expected, the best and nearly perfect agreement between the observed and predicted expression was obtained in the simulation for *MIR210HG* and the initial concentration of HIF1B in the simulation set to 60, i.e. for the initial conditions for which the model had been fitted. For the initial HIF1B concentration of 30, the induction of *MIR210HG* was approximately two-fold lower than observed experimentally, and for the initial HIF1B concentration set to 1 it was ~4 times lower. This comparison between the simulations for different initial values of HIF1B and the experiment for *MIR210HG* confirms that in our final model HIF1B is a limiting factor. For *EGLN3* the optimal, but not perfect, agreement was obtained for the initial HIF1B concentration of 30, with the lower initial concentration of 1 resulting in the induction smaller than observed and the higher initial concentration of 60 resulting in the induction higher than observed. For *BNIP3L* the optimal, very good agreement was obtained for the initial HIF1B concentration of 1, whereas the two higher concentrations of 30 and 60 resulted in the simulated induction stronger than observed. Our model predicted no induction for the highly induced *LUCAT1* gene with the total HRE count of 1, suggesting utilization of HRE sites in enhancers not included in our analysis. Thus, for the three useful genes, the optimal initial HIF1B concentrations were different for every gene, and lowest for the gene with the lowest total HRE count, and highest for the gene with the highest total HRE count. Thus, the simulation results for the three genes regulated by both HIF-1 and HIF-2 confirmed that in our ODE model the initial concentration of HIF1B can be a limiting factor for the induction of the target gene.

## Discussion

We developed an ODE model of hypoxia signaling and transcriptional activation of hypoxia responsive genes that takes into account not only HIF-1 but also HIF-2. Within this model, we were able to correctly simulate the effects of a further drop of oxygen level during hypoxia on the HIF switch. These simulations results supported experimentally established conclusion that residual PHD activity under hypoxia contributes to the HIF-switch [1]. By simulations in the model we found out that, for the simulation results to broadly agree with experiments, for the highly induced *MIR210HG* gene, there is a need for a large excess of HIF1B over the two HIF α subunits. Only after choosing the value of 60-fold excess (as compared to that of HIF1A), based on the simulations in our model taking into account both HIFs, we noticed that this value is about two times higher than the ratio of the initial concentrations of HIF1A and HIF1B (5 and 170, respectively) set in the model of Nguyen et al. (2013) [3]. The results of the simulations in our final model for additional genes, in particular for *BNIP3L*, for which the optimal HIF1B initial concentration was equal to that of HIF1A, demonstrate that in our ODE model the optimal HIF1B initial concentration is gene-dependent. This means that from the modeling results alone we cannot postulate what this concentration is inside the cell nucleus.

To the best of our knowledge, there are no published data available that present absolute quantification of HIF1A, HIF2A, and HIF1B (alias ARNT) simultaneously in a single cell line. Most studies focus on relative expression, transcriptional activity, or protein stabilization dynamics. The available experimental data comprise of our ELISA measurements in HUVECs reported herein for HIF1A (0.2 ng/mg of total protein under normoxia, 0.66 ng/mg of total protein after 8 h of hypoxia) and HIF2A (0.6 ng/mg of total protein under normoxia, 3.5 ng/mg of total protein after 8 h of hypoxia). The ELISA measurements of ARNT in hippocampal neurons [44] yielded 0.19 ng/mg of total protein under normoxia, 0.35 ng/mg of total protein after 18 h of hypoxia. Earlier measurements of ARNT in a number of mostly cancer cell lines by quantitative western blot (utilizing a standard curve), performed by Holmes and Pollenz (1997) [45], yielded normoxic ARNT concentrations in the range 6.6–21.3 ng/mg of total protein, that is ~30–60 times higher than the ELISA measurements in the hippocampal neurons. We speculate that the molar ratio of HIF1B to HIF α subunits is likely higher in the nucleus than in the whole cell, especially at the beginning of hypoxia, because the localization of HIF1B at both normoxia and hypoxia is predominantly nuclear [44, 46], while the newly stabilized HIF α subunits are initially cytoplasmic.

Our modeling results, in conjunction with the experimental data that HIF1B concentration varies substantially between different cell types [44, 45], are suggesting that HIF1B may be a limiting factor of the response to hypoxia, which we observed during the model fitting and in the simulations for additional genes in the final model*.* The hypothesis that HIF1B can be a limiting factor of the response to hypoxia was first put forward by Mandl et al. (2013) [33], to explain the findings that some cancer cell lines up-regulate HIF1B in response to hypoxia. This hypothesis is now supported by considerable experimental evidence, reviewed by Mandl and Depping (2017) [47]. In particular, Choi et al. (2006) [48] showed that induction of ARNT degradation blocked HIF-1 signaling. Overexpression of ARNT in Hep3B cells increased HRE-driven luciferase signal under hypoxia [49]. Sequestration of ARNT by HIF1A under hypoxia blocked AHR signaling [50, 51]. In the mouse a splice variant of HIF-3α competes for ARNT with HIF1A and HIF2A, and thus effectively suppresses their activity in endothelial cells [52]. Our model predicts a similar competition for HIF1B between HIF1A and HIF2A, which may contribute to the results of the HIF switch. Our modeling results furthermore suggest that modulation by the level of available HIF1B will affect especially the response to hypoxia of the genes utilizing many HRE sites strongly induced by hypoxia, such as *MIR210HG*.

The regulation of a given gene under hypoxic conditions is influenced not only by the presence of HRE motifs, but also by epigenetic mechanisms specific to the cell type/state, including the chromatin openness in the promoter region, as well as the interactions between promoters and enhancers [53, 54]. An important aspect of the work published in Cellular Signalling [2] and of the final ODE model in the current work has been the selection of open chromatin areas in gene promoters as the regions in which we counted HRE motifs. In this way, by integrating the genomic sequence (HRE motifs) with epigenomic information (chromatin openness), we were able to substitute the abstract HRE concentrations used in Nguyen et al. (2013) model [3], with counts of specific motifs in well-defined functional cis-regulatory regions. While we used the HRE counts in promoter open chromatin regions, our ODE model can be extended to the situation of known enhancer-promoter mappings, by summing the HRE counts in promoters and in enhancers, as is currently done for distinct open chromatin regions within a promoter. The usefulness of such extension would require testing when such mappings become available.

**Key Points**

- We developed a mathematical model of the response to hypoxia that takes into account not only HIF-1 but also HIF-2.
- The model helped to elucidate the mechanisms of the HIF switch during the response of Human Umbilical Endothelial Cells (HUVECs) to hypoxia.
- With the model we predict that HIF-1β may be a limiting factor of the response to hypoxia, especially of the genes utilizing many HRE sites strongly induced by hypoxia, such as *MIR210HG*.

## Supplementary Material

Supplementary_File1_rev1_v2_with_figure_elaf021

Hypoxia_model_elaf021

## Code availability

The full details of our model, including values of all parameters and the initial concentrations are provided in the software-readable SBML format (https://sbml.org/) as Supplementary File 2.
