# Correction to: A dynamic model of gene activation in response to hypoxia accounting for both HIF-1 and HIF-2

**DOI:** 10.1093/bfgp/elaf028

**Published:** 2025-12-23

This is a correction to: Aleksandra Cabaj, Agata Charzyńska, Adrianna Moszyńska, Maciej Jaśkiewicz, Rafał Bartoszewski, Michał Dąbrowski, A dynamic model of gene activation in response to hypoxia accounting for both HIF-1 and HIF-2, *Briefings in Functional Genomics*, Volume 24, 2025, https://doi.org/10.1093/bfgp/elaf028

In the originally version of the manuscript, only one of the two supplementary data files was published. The second has now been added to the article (Hypoxia_model_elaf021.xml).

