# Supplementary material for: A dynamic model of gene activation in response to hypoxia accounting for both HIF-1 and HIF-2
Source: Brief Funct Genomics. 2025 Dec 12;24:elaf021. doi: 10.1093/bfgp/elaf021 (PMC12700088; doi:10.1093/bfgp/elaf021)
Supplement: Supplementary_File1_rev1_v2_with_figure_elaf021 [file supplementary_file1_rev1_v2_with_figure_elaf021.pdf]

Supplementary File S1 to the publication: “A dynamic model of gene activation in response to hypoxia accounting for both HIF-1 and HIF-2”

by Aleksandra Cabaj[†], Agata Charzyńska[†], Adrianna Moszyńska, Maciej Jaśkiewicz, Rafał Bartoszewski, Michał Dąbrowski

[†] Joint first authors

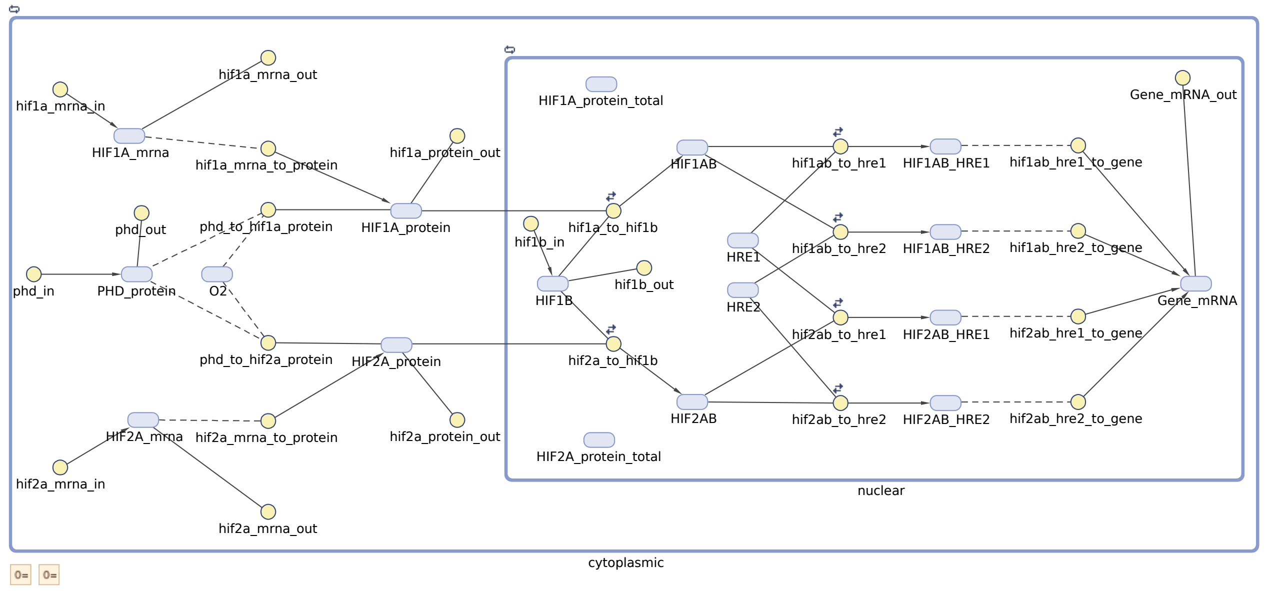

Supplementary Figure S1. The diagram of the full model.

Model diagram and description

Suppl. Fig. S1 shows the diagram of the full model. Blue ovals represent the species (e.g. HIF1A_mrna) and the yellow circles represent reactions between the species (e.g. hif1a_mrna_to_protein). All of the reactions are written in lowercase letters, and all of the species start with the name of the molecule written in capital letters. All of the species influxes contain the “_in” suffix, whereas outflows are marked by the “_out” suffix. As the diagram in Suppl. Fig. S1 shows from left to right, the influxes of HIF1A and HIF2A mRNAs that represent the transcription process of those species (hif1a_mrna_in and hif2a_mrna_in). The outflows of those species represent the degradation of mRNAs (hif1a_mrna_out and hif2a_mrna_out). The influx of PHD protein represents the translation process of this species (phd_in). The center of the diagram shows mRNA species being translated to HIF proteins (hif1a_mrna_to_protein and hif2a_mrna_to_protein) and the simplified process of HIF degradation due to oxygen-dependent PHD-mediated hydroxylation of those proteins (phd_to_hif1a_protein and phd_to_hif2a_protein). HIF proteins are also degraded by mechanisms other than PHD-mediated hydroxylation and degradation, which is represented by outflows of those proteins from the system (hif1a_protein_out and hif2a_protein_out). The inflow and outflow of HIF1B protein are given by hif1b_in and hif1b_out. HIF1B can form dimers with both HIF1A and HIF2A proteins. Free HIF1A and HIF2A subunits of HIFs bind with HIF1B subunit to form transcriptionally active heterodimers (HIFAB). We assumed that this reaction is reversible. These transcriptionally active heterodimers can reversibly bind to HRE elements in target gene promoters and enhancers, forming HIFAB-HRE complexes. Finally, we assumed that all HIF-HRE complexes can activate the transcription of the target gene.

Supplementary Table S1. Reactions, reaction rates (fluxes) and fitted parameter values of the final model fitted to *MIR210HG*.

No.	Reaction	Reaction rate	Parameter value[#]	
1	PHD_protein + HIF1A_protein + O_2 −> O_2 + PHD_protein	phd_to_hif1a_protein = phd_to_hif1a_protein.vm · O_2 / (phd_to_hif1a_protein.km + O_2) · PHD_protein · HIF1A_protein / (phd_to_hif1a_protein.nm + HIF1A_protein)	vm	3.51
			km	0.2266
			nm	6.6677
2	PHD_protein + HIF2A_protein + O_2 −> O_2 + PHD_protein	phd_to_hif2a_protein = phd_to_hif2a_protein.vm · O_2 / (phd_to_hif2a_protein.km + O_2) · PHD_protein · HIF2A_protein / (phd_to_hif2a_protein.nm + HIF2A_protein)	nm	7.6866
			km	0.31486
			vm	3.0278
3	null −> PHD_protein	phd_in = phd_in.kf	0.20707	
4	PHD_protein −> null	phd_out = phd_out.kf · PHD_protein	0.024154	
5	null −> HIF1A_mrna	hif1a_mrna_in = hif1a_mrna_in.kf	0.9	
6	HIF1A_mrna −> null	hif1a_mrna_out = hif1a_mrna_out.kf · HIF1A_mrna	1.6	
7	HIF1A_mrna −> HIF1A_mrna + HIF1A_protein	hif1a_mrna_to_protein = hif1a_mrna_to_protein.kf · HIF1A_mrna	5.4	
8	null −> HIF2A_mrna	hif2a_mrna_in = hif2a_mrna_in.kf	112.03	
9	HIF2A_mrna −> null	hif2a_mrna_out = hif2a_mrna_out.kf · HIF2A_mrna	137.43	
10	HIF2A_mrna −> HIF2A_mrna + HIF2A_protein	hif2a_mrna_to_protein = hif2a_mrna_to_protein.kf · HIF2A_mrna	12.619	
11	HIF2A_protein −> null	hif2a_protein_out = hif2a_protein_out.kf · HIF2A_protein	0.11987	
12	HIF1A_protein −> null	hif1a_protein_out = hif1a_protein_out.kf · HIF1A_protein	$5.0119 \cdot 10^{-06}$	
13	HIF2A_protein + HIF1B <−> HIF2AB	hif2a_to_hif1b = hif2a_to_hif1b.kf · HIF2A_protein · HIF1B − hif2a_to_hif1b.kd · HIF2AB	kf	0.027722
			kd	0.00023875
14	HIF1A_protein + HIF1B <−> HIF1AB	hif1a_to_hif1b = hif1a_to_hif1b.kf · HIF1A_protein · HIF1B − hif1a_to_hif1b.kd · HIF1AB	kf	0.0095423
			kd	0.00011719
15	HIF2AB + HRE2 <−> HIF2AB_HRE2	hif2ab_to_hre2 = hif2ab_to_hre2.kf · HIF2AB · HRE2 − hif2ab_to_hre2.kd · HIF2AB_HRE2	kf	7.2823
			kd	0.010149
16	HIF2AB + HRE1 <−> HIF2AB_HRE1	hif2ab_to_hre1 = hif2ab_to_hre1.kf · HIF2AB · HRE1 − hif2ab_to_hre1.kd · HIF2AB_HRE1	kf	16.936
			kd	$1.3126 \cdot 10^{-10}$
17	HIF1AB + HRE2 <−> HIF1AB_HRE2	hif1ab_to_hre2 = hif1ab_to_hre2.kf · HIF1AB · HRE2 − hif1ab_to_hre2.kd · HIF1AB_HRE2	kf	0.64502
			kd	0.00084244
18	HIF1AB + HRE1 <−> HIF1AB_HRE1	hif1ab_to_hre1 = hif1ab_to_hre1.kf · HIF1AB · HRE1 − hif1ab_to_hre1.kd · HIF1AB_HRE1	kf	1.2738
			kd	0.0002167
19	HIF2AB_HRE2 −> Gene_mRNA + HIF2AB_HRE2	hif2ab_hre2_to_gene = hif2ab_hre2_to_gene.kf · HIF2AB_HRE2	1.4481	
20	HIF2AB_HRE1 −> Gene_mRNA + HIF2AB_HRE1	hif2ab_hre1_to_gene = hif2ab_hre1_to_gene.kf · HIF2AB_HRE1	0.40006	
21	HIF1AB_HRE2 −> Gene_mRNA + HIF1AB_HRE2	hif1ab_hre2_to_gene = hif1ab_hre2_to_gene.kf · HIF1AB_HRE2	1.247	
22	HIF1AB_HRE1 −> Gene_mRNA + HIF1AB_HRE1	hif1ab_hre1_to_gene = hif1ab_hre1_to_gene.kf · HIF1AB_HRE1	0.26598	
23	Gene_mRNA −> null	Gene_mRNA_out = Gene_mRNA_out.kf · Gene_mRNA	0.53854	
24	null −> HIF1B	hif1b_in = hif1b_in.kf	0.63248	
25	HIF1B −> null	hif1b_out = hif1b_out.kf · HIF1B	0.45914	

[#] The units of time are hours, the concentrations are expressed as relative units.
